# A Novel and Fast Encryption System Based on Improved Josephus Scrambling and Chaotic Mapping

**DOI:** 10.3390/e24030384

**Published:** 2022-03-09

**Authors:** Zhaoxiong Guan, Junxian Li, Linqing Huang, Xiaoming Xiong, Yuan Liu, Shuting Cai

**Affiliations:** 1 School of Automation, Guangdong University of Technology, Guangzhou 510006, China; 2112004240@mail2.gdut.edu.cn (Z.G.); 3219008903@mail2.gdut.edu.cn (J.L.); xmxiong@gdut.edu.cn (X.X.); eeliuyuan@gdut.edu.cn (Y.L.); 2 School of Advanced Manufacturing, Guangdong University of Technology, Guangzhou 510006, China

**Keywords:** Josephus ring, image cryptosystem, scrambling framework, plaintext-related, chaotic mapping, efficiency

## Abstract

To address the shortcomings of weak confusion and high time complexity of the existing permutation algorithms, including the traditional Josephus ring permutation (TJRP), an improved Josephus ring-based permutation (IJRBP) algorithm is developed. The proposed IJRBP replaces the remove operation used in TJRP with the position exchange operation and employs random permutation steps instead of fixed steps, which can offer a better scrambling effect and a higher permutation efficiency, compared with various scrambling methods. Then, a new encryption algorithm based on the IJRBP and chaotic system is developed. In our scheme, the plaintext feature parameter, which is related to the plaintext and a random sequence generated by a chaotic system, is used as the shift step of the circular shift operation to generate the diffusion matrix, which means that a minor change in the source image will generate a totally different encrypted image. Such a strategy strikes a balance between plaintext sensitivity and ciphertext sensitivity to obtain the ability to resist chosen-plaintext attacks (CPAs) and the high robustness of resisting noise attacks and data loss. Simulation results demonstrate that the proposed image cryptosystem has the advantages of great encryption efficiency and the ability to resist various common attacks.

## 1. Introduction

### 1.1. Research Background

With the development of communication technology, some new transmission media such as image and video are widely used to disseminate information. Digital images containing private information without special processing can be easily intercepted and exploited by hackers when they are transmitted on various public channels. Encryption is an effective means commonly used to keep information confidential. However, traditional text encryption standards such as the data encryption standard (DES), advanced encryption standard (AES) and Rivest–Shamir–Adleman (RSA) algorithm cannot efficiently encrypt the images with huge data volumes, high temporal redundancy, and spatial redundancy [1]. The high sensitivity to initial values and the uncertain behavior of chaotic systems renders them more suitable for image encryption [2]. Therefore, the chaotic-based encryption scheme draws more and more attention. In 1998, Fridrich proposed a new cryptographic framework that includes permutation and diffusion using a two-dimensional chaotic map. Such a method not only disrupts the position of the pixels, but also changes the statistical characteristic of the plaintext image [3]. Inspired by Fridrich’s research, many encryption algorithms using similar encryption frameworks have been proposed in recent years [1,4,5,6,7,8,9,10,11,12,13,14,15,16,17,18,19,20,21,22,23,24,25,26,27,28,29,30,31].

According to the characteristics of the scrambling method, the above algorithms can be divided into two categories: one is that the size of the original image remains unchanged [1,4,5,6,7,8,9,10,11,12,13,14,19,23,28,29], and the other is that the plaintext image is decomposed into another size for scrambling [15,16,17,18,20,21,22,24,25,26,27].

In the first category, some researchers utilized the extended Arnold map to permute the plaintext image [6,9]. Specifically, the pixel coordinates are set as the initial values of the chaotic map and the new positions of the pixels are obtained by iterating the chaotic system. To improve the plaintext sensitivity of a cryptosystem, in [6], the plaintext information is employed to generate the parameters of the extended Arnold map and influence the diffusion operation. Kang et al. [7] presented a novel plaintext-related mechanism, in which the numbers of A, T, C, and G of plaintext DNA coding are used to control the permutation process. In addition, SHA algorithms have been applied in cryptographic frameworks to pursue high plaintext sensitivity [8,11,12,14,23]. For instance, in [8], the hash values of the plaintext image calculated by the SHA256 algorithm are used as the initial values of the non-coupled map lattice function. However, these schemes suffer from some drawbacks, such as the weak scrambling effect [6,9], a long permutation time [7], or a low application value because of its one-time-pad-like property [8,11,12,14,23]. For the characteristic of easy implementation, permutations based on index matrices which are derived from sorting and matching random sequences has been used in many encryption schemes [4,5,28,29]. In 2020, Cao et al. [5] employed the index matrix and the specific diffusion formula to permute and diffuse the plaintext image separately, which can avoid the need of using one-time encryption techniques to reach high plaintext sensitivity. In many works, circular shift is exploited to scramble the source image matrix to achieve faster permutation speeds [1,10,13]. In [1], a novel cryptosystem is developed based on a circular shift operation in which step size is controlled by the pseudo-random sequence. Although this scheme has excellent encryption efficiency, its scrambling effect needs to be further improved.

In the second category, the permutation operation in many cryptographic systems is performed on a one-dimensional plaintext matrix transformed from the original image [16,17,20,24,26]. For instance, in [17], the plain image is converted into a one-dimensional matrix, which is scrambled by an index matrix in the permutation stage. Since a pixel can be represented by an eight-bit binary, some works transform the original image into a binary array which is downscaled to a one-dimensional matrix for further scrambling [20,24]. Although bit-level permutation can change the positions and values of pixels simultaneously, the amount of data that needs to be processed has increased by 8 times, which reduces the efficiency of the cryptographic system. Furthermore, since the generation of a index matrix used in permutation stage by sorting and comparing the random sequences is very time-consuming, and when the size of the original image is doubled, the execution time will increase exponentially, some algorithms transformed the plain image into a 3D matrix for further processing [15,25,27]. In 2016, Zhang et al. proposed a novel cryptosystem in which the plaintext image is transformed into a 3D bit matrix, and then the permutation operation is performed using three index sequences whose lengths are equal to the length, width, and height of the 3D bit matrix [15], respectively. In 2014, a Josephus ring has been used to scramble images [32]. However, this exposed some problems, such as a fixed step length and a too-long scrambling time. In recent years, many improved Josephus rings have been developed and used to pursue higher confusion effects [18,21,22]. In [18], Niu et al. developed an improved Josephus ring scrambling algorithm with a dynamic step size, related to the pixel value of the plaintext. This scheme greatly improves the scrambling effect and plaintext sensitivity, but possesses poor robustness against noise attacks and a slow encryption speed. In addition to the permutation–diffusion architecture, some scholars apply quantum mechanics theory to developed image encryption schemes which only contain some diffusion operations, such as C-Not gate [33,34]. Other studies introduced additional perturbations to the chaotic maps to avoid chaos degradation [35] when implementing in hardware with limited precision [36,37].

### 1.2. The Weaknesses of Existing Works

After careful analysis of the above encryption schemes, we found that most of the algorithms have the following drawbacks:

- Some encryption schemes are insensitive to subtle differences of the original image and insufficient to resist chosen-plaintext attacks (CPAs). Table 1 shows the papers that have been cracked in recent years;
- Low robustness of noise-resistings and occlusion-resistings because of the high sensitivity of ciphertext [7,16,18,38];
- Permutation operations are time-consuming, especially the permutation techniques using long random sequences generated by sorting and comparing operations [4,5,11,12,17,19,20] or traditional Josephus rings [18,21,22];
- Poor permutation effects are present in many works, including existing chaos-based image permutation algorithms and Josephus ring-based permutations, which is discussed in Section 1 and will be further detailed in Section 3.

### 1.3. Contribution of Our Research

To overcome the weaknesses of existing works, we propose an improved Josephus ring-based permutation (IJRBP) and a new encryption scheme. The contributions of this paper are as follows:

- The proposed IJRBP replaces the remove operation used in TJRP with the position exchange operation and employs random permutation steps instead of fixed steps, which avoids the drawbacks of TJRP to offer an excellent scrambling effect and a high permutation efficiency;
- A new encryption algorithm based on the IJRBP is developed. The new scheme strikes a balance between plaintext sensitivity and ciphertext sensitivity to obtain the ability to resist CPAs, as well as a high robustness for resisting noise attacks and data loss simultaneously;
- IJRBP can be used for scrambling grayscale images or color images of any size;

Section 2 presents the involved chaotic systems and the generation of pseudo-random sequences required for cryptosystems. Section 3 introduces the IJRBP algorithm in detail. Section 4 provides the process of image encryption. Section 5 offers a systematic evaluation of safety performance. Section 6 concludes this article.

## 2. The Generation of a Pseudo-Random Sequence

### 2.1. The Involved Chaotic Map

There are three chaotic maps used in this work, namely, a tent map, a piecewise linear map, and a Chebyshev map. The tent map is a classic one-dimensional chaotic system which is widely used in the field of image encryption, and its mathematical equation can be defined as:

(1) $$x_{n+1}=F_{1}\left(x_{n},u\right)=\left\{\begin{array}{cc} \frac{ux_{n}}{2} & x_{n}<0.5\\ \frac{u\left(1-x_{n}\right)}{2} & x_{n}\geq 0.5; \end{array}\right.$$

when the control parameter $u\in \left(2,4\right]$, the numerical simulation of the tent map demonstrates chaotic behavior.

The piecewise linear map consists of a multi-segment linear function and can be described by the following iteration:

(2) $$x_{n+1}=F_{2}\left(x_{n},p\right)=\left\{\begin{array}{c} x_{n}/p,0<x_{n}<p\\ \left(x_{n}-p\right)/\left(0.5-p\right),p<x_{n}<0.5\\ F\left(1-x_{n},p\right),0.5<x_{n}<1, \end{array}\right.$$

where *p* is the control parameter in the range $\left(0,0.5\right)$ and the $x_{n+1}\in \left(0,1\right)$ is the output.

The Chebyshev map is a one-dimensional chaotic map with the advantages of a simple structure and easy implementation. It can be expressed as:

(3) $$x_{n+1}=F_{3}\left(x_{n},a\right)=\cos\left(a\times \arccos x_{n}\right),$$

where a≥2 and $x_{n+1}\in \left[-1,1\right]$.

**Figure 1:**
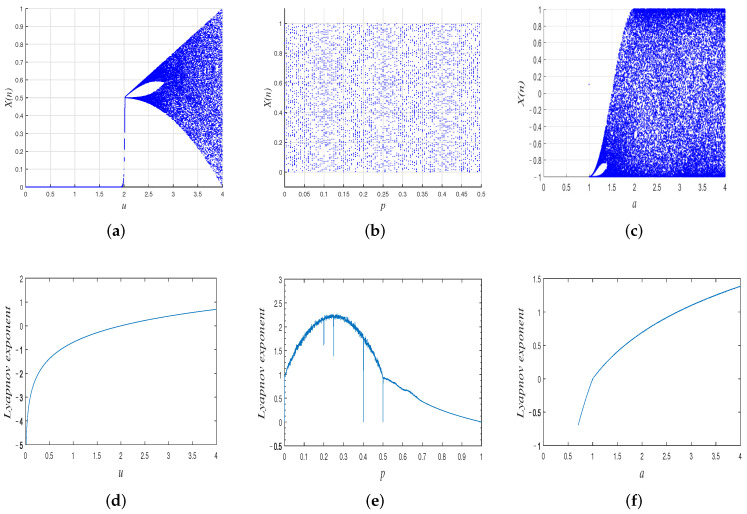
(**a**–**c**) are the bifurcation diagrams of the tent map, piecewise linear map, and Chebyshev map, respectively; (**d**–**f**) are the corresponding Lyapunov exponent diagrams.

Figure 1 shows the bifurcation diagrams and Lyapunov exponent diagrams of the chaotic maps used in the proposed cryptosystem. In our proposed scheme, the parameters of the three chaotic maps are set as u=3.999998, a=4, and p=0.256, respectively.

### 2.2. Pseudo-Random Sequence Generation

In this subsection, the three involved chaotic maps are used to generate the pseudo-random sequences which will be utilized in the proposed cryptosystem.

Step 1: An original image *I* is converted into a one-dimensional matrix *P* with the length of MN, where *M* and *N* are the height and width of the image. Iterate Equations (1)–(3) $\left(N_{0}+M\right)$, $\left(N_{0}+N+2\right)$, and $\left(N_{0}+M\right)$ times, respectively, and discard the first $N_{0}$ elements for a better random effect and to obtain three sequences $x_{n}$, $y_{n}$, and $z_{n}$, given by:

(4) $$\left\{\begin{array}{c} x_{n}=\left\{x_{1},x_{2},x_{3},\ldots ,x_{M}\right\}\\ y_{n}=\left\{y_{1},y_{2},y_{3},\ldots ,y_{N+2}\right\}\\ z_{n}=\left\{z_{1},z_{2},z_{3},\ldots ,z_{M}\right\} \end{array}\right.$$

Step 2: Obtain the two sequences Xn and Zn calculated by Equations (5) and (6):

(5) $$Xn\left(k\right)=floor\left(mod\left(x_{n}\left(i\right)\times y_{n}\left(j\right)\times 10^{9},M*N\right)\right)+1,$$

(6) $$Zn\left(k\right)=floor\left(mod\left(y_{n}\left(i\right)\times abs\left(z_{n}\left(j\right)\right)\times 10^{9},256\right)\right),$$

where i=1,2,3⋯M,j=1,2,3⋯N, and k=1,2,3⋯MN.

Step 3: Generate the coordinate information. The random coordinates loc1 and loc2 are calculated by using the following equations:

(7) $$loc1=floor\left(mod\left(y_{n}\left(N+1\right)\times 10^{9},M\right)\right)+1,$$

(8) $$loc2=floor\left(mod\left(y_{n}\left(N+2\right)\times 10^{9},N\right)\right)+1.$$

Step 4: Obtain the plaintext feature parameter *f* using the sequence Xn and the plaintext image *I*, given by:

(9) $$\begin{array}{c} Xn_{1}=reshape\left(Xn,\left[M,N\right]\right), \end{array}$$

(10) $$\begin{array}{c} f=mod(\sum_{i=1}^{M}\sum_{j=1}^{N}\left(I\left(i,j\right)\times floor\left(\sqrt{Xn_{1}\left(i,j\right)}\right)\right),256), \end{array}$$

where i=1,2,3⋯M, and j=1,2,3⋯N.

## 3. Improved Josephus Ring-Based Permutation

**Figure 2:**
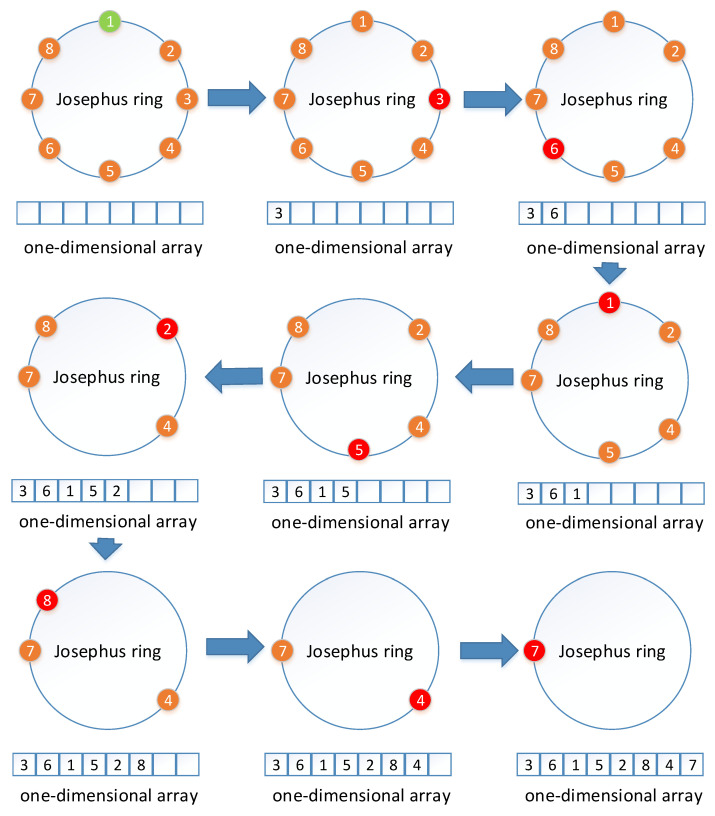
An illustration of Josephus ring with a step size of 3.

**Figure 3:**
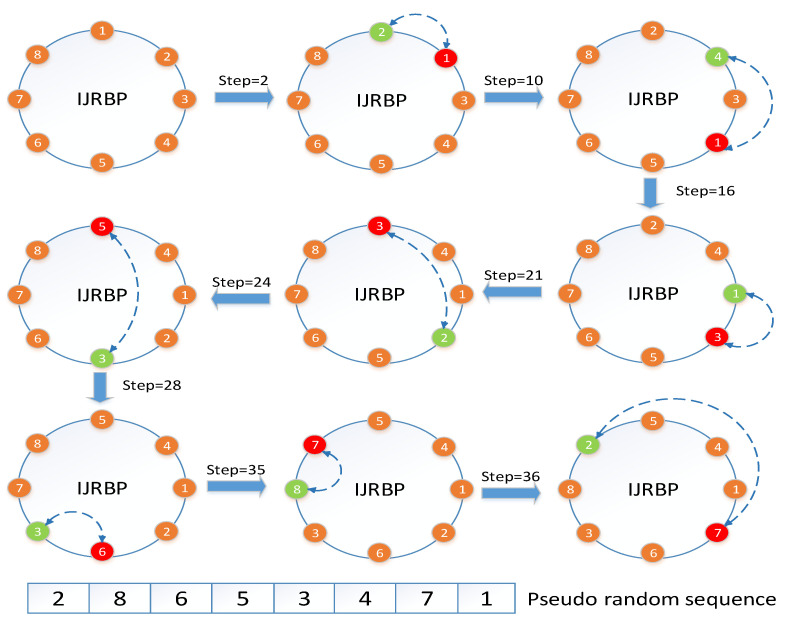
An illustration of IJRBP.

The traditional Josephus ring can be implemented with only one step parameter, and its permutation process is easy to understand. The principle of traditional Josephus rings is shown in Figure 2. Here, the step size is set to three and the Josephus ring is scanned clockwise from the first element. The element located at each step is extracted from the Josephus ring, and then a new similar operation is repeated from the next position of the removed element until the last element in the Josephus ring is eliminated. We improve the traditional Josephus ring and propose IJRBP to solve the problems of the traditional Josephus ring and other existing permutation algorithms discussed in Section 1. The principle of IJRBP is shown in Figure 3. The IJRBP replaces the remove operation with the position exchange operation to achieve a further dislocation effect and reduce the time complexity. Suppose the one-dimensional image matrix and the random sequence are P=[1,2,3,4,5,6,7,8] and Seq=[2,8,6,5,3,4,7,1], respectively. The detailed process of IJRBP is described in Algorithm 1.

**Figure 4:**
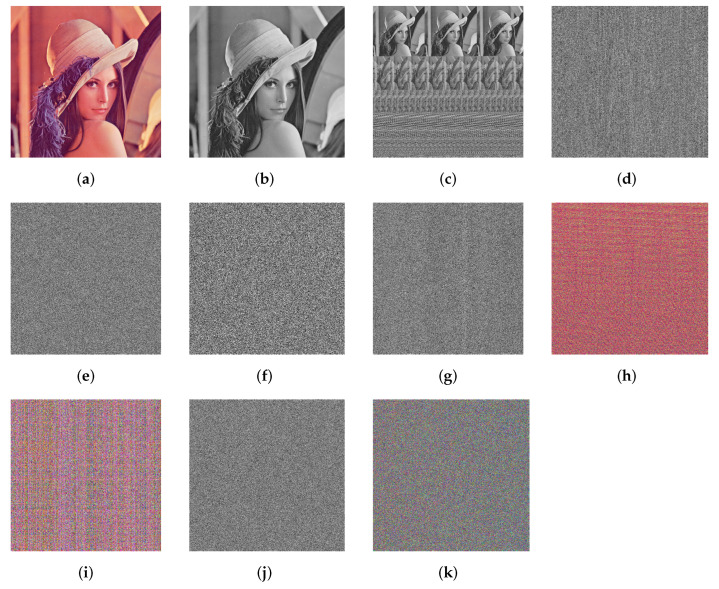
Scrambling effects of different schemes: (**a**,**b**) source images; (**c**–**i**) the permuted images are acquired by a traditional Josephus ring with a step size of 3 [1,5,6,18,24,38]; (**j**,**k**) the permuted images are acquired by IJRBP.

We use the standard testing image Lena in the scrambling experiment. All permutation algorithms are tested in the same environment to ensure the correctness. The simulation results are shown in Figure 4 and Table 2, and we can see that IJRBP shows significant advantages over other solutions in terms of the confusion effect and the running speed.
**Algorithm 1:** Pseudo-code of IJRBP**Input:** The plaintext image *P* with size of M×N, a random sequence Xn with length MN**Output:** output result permuted plaintext matrix *S*.1:Convert *P* into a one-dimensional matrix P1 with length MN.2:cd=0,occupied=0,posi=0;3:**for**i=1:MN**do**4:      temp=P1(i);5:      cd=cd+Xn(i);6:    **if** MN−occupied<cd **then**7:            posi=cd−(MN−occupied);8:            posi=mod(posi,MN);9:        **if** posi==0 **then**10:                  posi=MN;11:        **end if**12:    **else**13:            posi=cd+occupied;14:    **end if**15:      P1(i)=P1(posi);16:      P1(posi)=temp;17:      occupied=posi;18:**end for**19:Convert P1 to a two-dimensional matrix *S* with size of M×N.

## 4. The Proposed Encryption System

**Figure 5:**
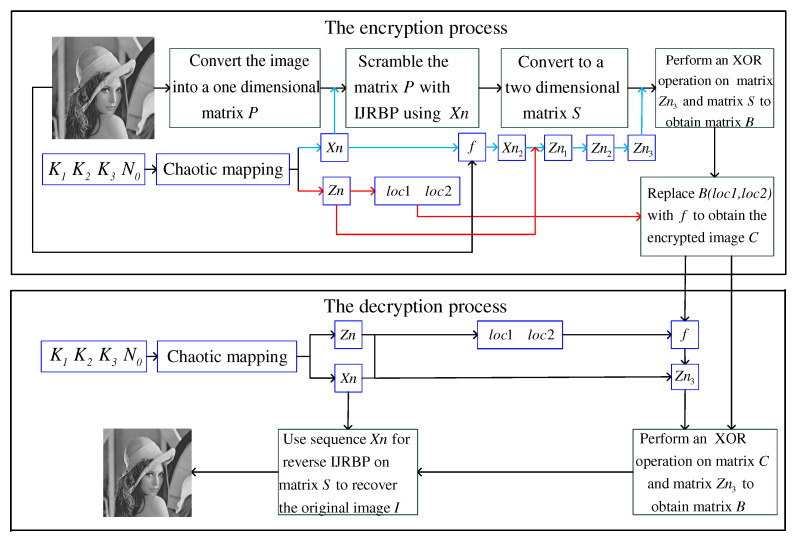
The proposed cryptosystem.

In this section, the new encryption system based on IJRBP is detailed. Figure 5 describes the diagram of the encryption and decryption system. After the IJRBP, the scrambled matrix *S* is obtained. It is worth noting that when the plaintext image *I* is all black, matrix *S* is obtained by Equation (11):

(11) $$S=mod\left(Xn_{1}+I,\;256\right).$$

### 4.1. Diffusion Stage

Here, we detail the diffusion stage as follows.

Step 1: Generate a pseudo-random sequence $Xn_{2}$ through Equation (12). Then, the IJRBP and $Xn_{2}$ are used to confuse the sequence Zn to obtain sequence $Zn_{1}$.

Step 2: Apply the circular shift operation on the sequence $Zn_{1}$ to gain another sequence $Zn_{2}$. Technically, if the parameter *f* is an odd number, the direction of the cyclic shift is to the left, with a step size of floor(1.1f); inversely, the direction is to the right with the same step size.

(12) $$Xn_{2}=\mathrm{mod}(Xn*\left(f+1\right),M*N).$$

Step 3: Convert the sequence $Zn_{2}$ into a two-dimensional matrix $Zn_{3}$ with height *M* and length *N*.

Step 4: Perform the XOR operation of matrix $Zn_{3}$ and matrix *S* to obtain matrix *B*.

Step 5: Replace the pixel value of B(loc1,loc2) with *f*; then, an encrypted image *C* is obtained. In particular, for encrypting a color image *I*, we can perform encryption on the R, G, and B channel images separately in the same way.

### 4.2. Decryption Algorithm

Referring to Figure 5, the decryption process is the inverse process of encryption. Firstly, the four keys are used to generate the sequences Xn and Zn, and then the parameter *f* is obtained through loc1 and loc2. Secondly, after the IJRBP, circular shift, and XOR operation, the matrix $Zn_{3}$ and matrix *S* are derived. Finally, the original image *I* is produced by performing the inverse IJRBP on the matrix *S* using the sequence Xn.

## 5. Simulation Results and Security Analysis

**Figure 6:**
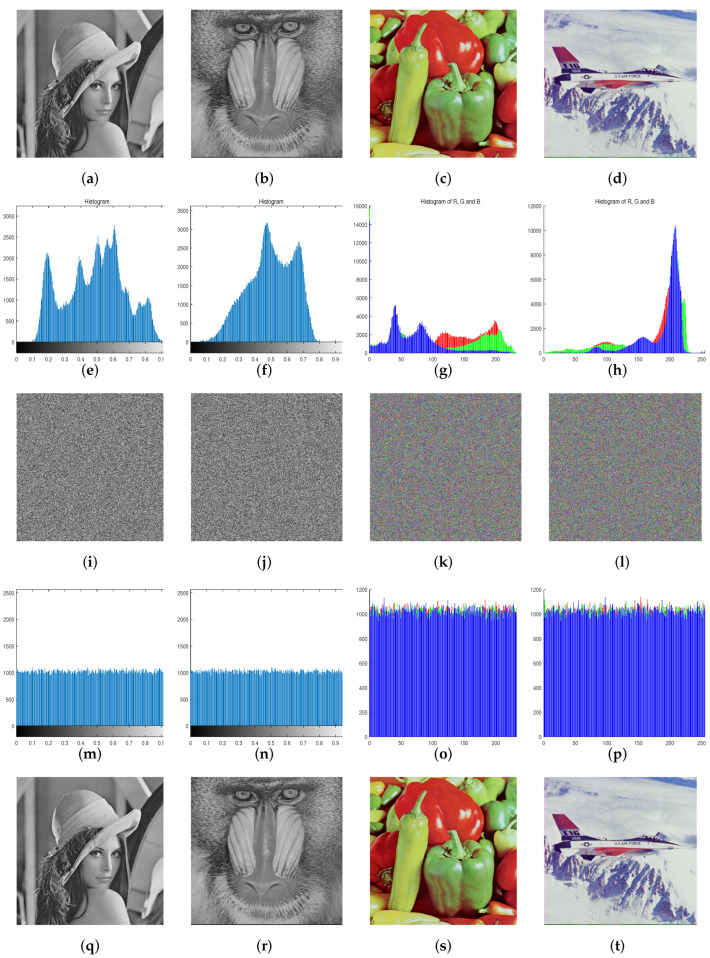
Simulation outcomes: (**a**–**d**) source images; (**e**–**h**) histograms of the source images; (**i**–**l**) cipher images of (**a**–**d**); (**m**–**p**) histograms of (**i**–**l**); (**q**–**t**) decrypted images of (**i**–**l**).

To verify the security and efficiency of the proposed encryption scheme, some standard images with different sizes are used in multiple simulation and security analyses. The initial values of the three chaotic maps, which are denoted as $K_{1}$, $K_{2}$, and $K_{3}$, and the iteration parameter $N_{0}$ are used as secret keys in this work. The simulation test is performed on a computer with an Intel Core i5-4200HCPU@ 2.80 GHz, 8.0G RAM, Windows 10 OS, and MATLAB R2016b. The simulation results are shown in Figure 6.

The peak signal to noise ratio (PSNR) is often used to measure the degree of signal distortion. A smaller PSNR means that the encrypted image possesses higher distortion relative to the original image. The PSNR is defined by:

(13) $$PSNR=10\times \log\frac{255^{2}}{MSE}\left(\mathrm{dB}\right),$$

where $MSE=\frac{1}{M\times N}\sum_{i=1}^{M}\sum_{j=1}^{N}{\left(I(i,j)-C(i,j)\right)}^{2}$, *M*, and *N* are the height and width of the image, and *I* and *C* are the plaintext image and the encrypted image, respectively. Table 3 shows the PSNR analysis results of different algorithms, which proves the excellent encryption effect of our algorithm.

### 5.1. Security Key Space

The key space is an important factor of a reliable encryption system and it must be greater than $2^{100}$ for resisting brute-force attacks. The ranges of the four secret keys in our approach are $K_{1}\in \left(0,1\right)$, $K_{2}\in \left(0,1\right)$, $K_{3}\in \left(-1,1\right)$, and $N_{0}\in \left[1000,2500\right]$, respectively. The key space comparison results of different algorithms are shown in Table 4. If the computational precision of the computer reaches $10^{16}$, the key space of the proposed algorithm will be $10^{16}\times 10^{16}\times 10^{16}\times 1500\approx 2^{170}$. Obviously, the cryptosystem can effectively resist brute-force attacks.

### 5.2. Histogram Analysis

Statistical analysis can disclose the distribution characteristics of the image and be used in the work of cracking cryptographic systems. An excellent encryption system must guarantee the uniform distribution of the pixel values of ciphertext image to mask the pixels’ distribution characteristics. As illustrated in Figure 6, the unique pixel intensity distribution characteristic of each plaintext image is concealed after the encryption operation. Furthermore, we use the variance of image histogram (VIH) to evaluate the flat level of the histogram of the ciphered image, which is defined as:

(14) $$VIH=\frac{1}{256}\sum_{i=0}^{255}(h_{i}-e{)}^{2},$$

where $h_{i}$ are the components of the histogram of the encrypted image and $e=\frac{M\times N}{256}$ (*M* and *N* are the size of image). Table 5 shows the VIH analysis results. Combining Figure 6 and Table 5, one can conclude that the VIH performance of the proposed scheme is better than that of other algorithms, and it is difficult for attackers to crack the encryption system through statistical analysis.

### 5.3. Correlation Analysis

**Figure 7:**
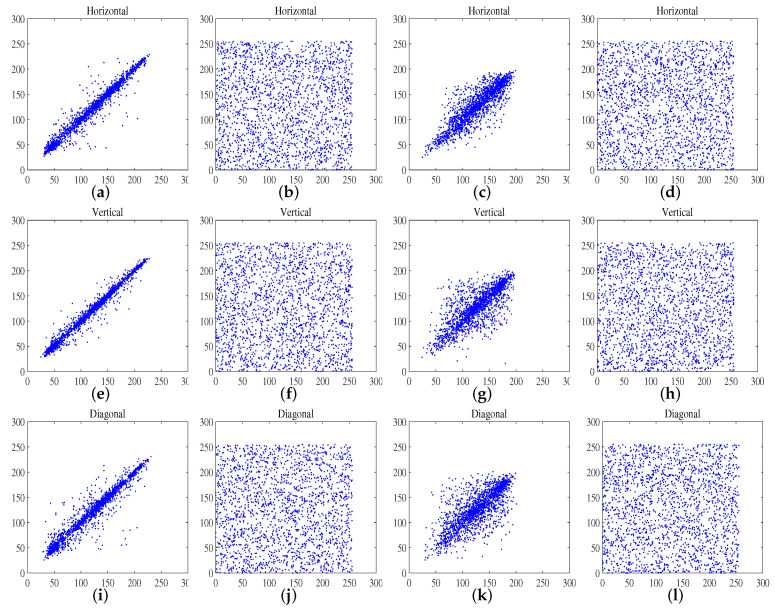
Correlation analysis. (**a**,**e**,**i**) are the correlation plots of the plain image of Lena; (**b**,**f**,**j**) are the correlation plots of the ciphered image of Lena; (**c**,**g**,**k**) are the correlation plots of the plain image of the baboon; (**d**,**h**,**l**) are the correlation plot of the ciphered image of the baboon.

There is a high correlation between the adjacent pixels in the image without special processing. The strong correlation of the adjacent pixels in cipher images will increase the risk of being attacked. Here, we calculate the correlation coefficient of all adjacent pixels at vertical, horizontal, and diagonal directions. The expressions of the correlation coefficient are defined as follows:

(15) $$r_{xy}=\frac{\mathrm{cov}(x,y)}{\sqrt{D(x)}\times \sqrt{D(y)}},$$

(16) $$\mathrm{cov}\left(x,y\right)=\frac{1}{N}\sum_{i=0}^{N}(x_{i}-E\left(x\right))\left(y_{i}-E\left(y\right)\right),$$

where *x* and *y* are the two adjacent pixel values, and *N* is the number of image pixels. Figure 7 shows the correlation plots of the Lena and Baboon images and the corresponding cipher images. Table 6, Table 7 and Table 8 present the correlation coefficient results, and one can see that the correlation coefficient of the image encrypted by the proposed algorithm is close to zero. Further, the average analysis results of our scheme is lower than that of other techniques.

### 5.4. Secret Key and Plaintext Sensitivity Analysis

#### 5.4.1. Secret Key Sensitivity Analysis

The high key sensitivity of a secure cryptosystem represents the excellent performance against exhaustive attacks. In this subsection, the NPCR (number of pixels change rate) and UACI (unified average changing intensity) are introduced to evaluate the key sensitivity and plaintext sensitivity. NPCR and UACI are defined by the following equation:

(17) $$\left\{\begin{array}{c} NPCR=\sum_{i=0}^{H}\sum_{j=0}^{W}D(i,j)\times 100\%\\ UACI=\frac{1}{W\times H}\sum_{i=0}^{H}\sum_{j=0}^{W}\frac{\left|C1(i,j)-C2(i,j)\right|}{255}\times 100\%, \end{array}\right.$$

where C1,C2 are two cipher images, and $D\left(i,j\right)=\left\{\begin{array}{c} 0,\;if\;C1(i,j)=C2(i,j)\\ 1,\;if\;C1(i,j)\neq C2(i,j) \end{array}\right.$.

Here, a simulation example is given, and its detailed steps are as follows:

Step 1: A secret key K1(0.2,0.4,0.3,2000) is selected from the key space and used to encrypt the original image of Lena to obtain the cipher image denoted by C1.

Step 2: Add $10^{-14}$ to the first initial value of K1 to obtain another secret key $K2(0.2+10^{-14},0.4,0.3,2000)$. Then, the modified key K2 is used to encrypt the same original image to obtained another cipher image, denoted as C2.

Step 3: Finally, we calculate the NPCR and UACI of C1 and C2, according to Equation (17).

We randomly select 200 sets of keys from the key space to repeat the above steps 200 times and the average results of NPCR and UACI are shown in Table 9. The numerical results of NPCR and UACI in Table 9 are the approximate theoretical values, which demonstrate that the encryption mechanism is extremely sensitive to the encryption keys.

#### 5.4.2. Plaintext Sensitivity Analysis

Differential attacks are the common methods used in cryptanalysis by attackers. By changing the pixel value of the plaintext image and recording the change of the corresponding ciphertext image, attackers may deduce the correspondence between the original image and the encrypted image or the equivalent keys. High plaintext sensitivity can ensure the ability of the encryption algorithm to resist differential attacks effectively. Here, we use NPCR and UACI again to test the plaintext sensitivity of the proposed scheme. In this experiment, we use 100 sets of keys to encrypt the original images and the same images with one pixel at a random position, slightly modified by Equation (18).

(18) $$Pixel\left(x_{i},y_{j}\right)=mod\left(Pixel\left(x_{i},y_{j}\right)+1,256\right).$$

The simulation results of NPCR and UACI are shown in Table 10, and are all close to the ideal values, which proves that our cryptosystem is sensitive to slight differences of the image and can resist differential attacks.

### 5.5. Resistance to Chosen Plaintext Attack Analysis

**Figure 8:**
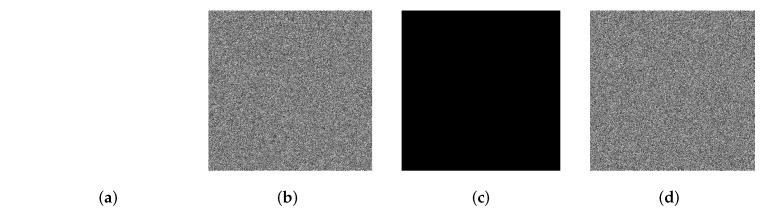
Simitation results of special images. (**a**) All white; (**b**) encrypted image of (**a**); (**c**) all black; (**d**) encrypted image of (**c**).

Furthermore, in a CPA, specially processed images, such as all black and all white images, are used to access the cryptosystem to obtain corresponding encrypted images for further cryptanalysis. In our scheme, to resist the CPA, the plaintext feature parameter *f*, which is calculated by Equation (10), is used to determine the shift step of the circular shift operation to generate the diffusion matrix, which guarantees the high plaintext sensitivity of the proposed algorithm. Here, four special images (P1,P2,P3,P4) were designed for this trial. P1 and P2 are all white and all black images, respectively. P3 is an image with only one pixel value of 1; the other pixel values are 0. P4 is an image with only one pixel value of 0, and the other pixel values are 255. The simulation results of the NPCR and UACI analyses and the encryption are shown in Table 11 and Figure 8, respectively. Based on the experimental results, the proposed encryption scheme has high plaintext sensitivity.

### 5.6. Information Entropy Analysis

Information entropy analysis can be used to reflect the degree of randomness of an encrypted image. The mathematical expression of information entropy is given by:

(19) $$H=\sum_{i=0}^{2^{N}-1}p(i)\log\frac{1}{p(i)},$$

where $p\left(i\right),i=1,2\cdots ,2^{N}$ is the probability of different gray-level values. According to Equation (19), the entropy value of a completely random grayscale image is 8. Table 12 shows the information entropy analysis results of original images and the cipher images encrypted with different schemes, which shows the better performance of our scheme than similar algorithms.

### 5.7. Noise Attack and Data Loss Analysis

**Figure 9:**
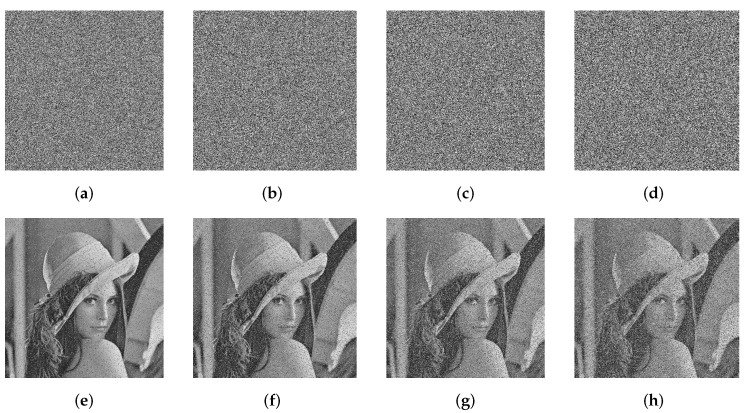
The recovery results of images attacked by noise: (**a**–**d**) are encrypted images affected by salt-and-pepper noise at densities of 0.1, 0.2, 0.3, and 0.4; (**e**–**h**) are the corresponding decrypted images.

**Figure 10:**
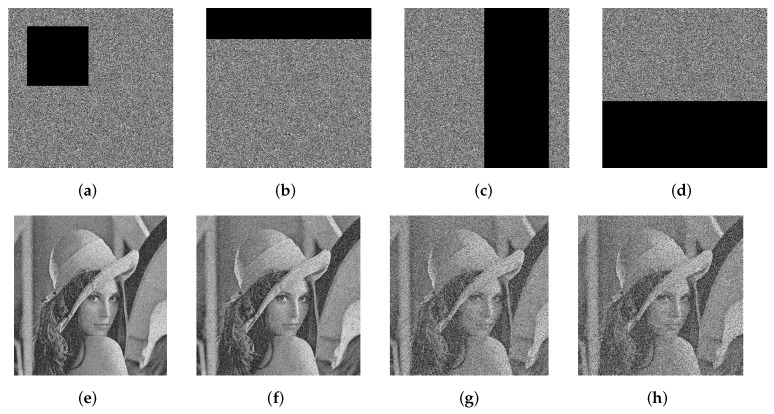
Simulation results of data loss: (**a**–**d**) are the encrypted images of Lena with different degrees of data loss; (**e**–**h**) are the corresponding decrypted images.

During the transmission to the receiver, the cipher image is easily affected by the harsh environment and the ability to recover the original image is lost. A reliable encryption scheme must minimize the impact of noise attacks and data loss. Here, a grayscale image of Lena with the size of 512×512 is selected to test the robustness of the proposed encryption algorithm to resist the noise attacks and data-loss attacks. The detailed analysis results are shown in Figure 9 and Figure 10. One can see that, even if the ciphertext images are polluted by salt-and-pepper noise with a noise intensity level of 0.4, most of the important information in the original images can still be obtained from the decrypted images. Therefore, our algorithm possess strong robustness in resisting noise attacks or data loss.

Furthermore, we use the PSNR again to quantify the robustness analysis of our cryptosystem. The analysis results are presented in Table 13 and attest that the encryption system has better recovery capability for the polluted information.

### 5.8. Encrypted Time Analysis

**Figure 11:**
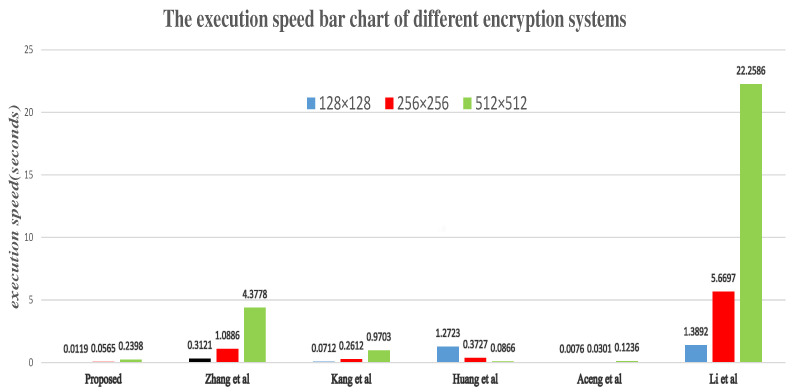
The execution speed bar chart of different encryption systems.

For the purpose of real-time encryption, the cryptosystem must have low computational complexity. The encryption speed simulation of our scheme and of similar recently proposed algorithms is performed in the same environment. Here, the works of Zhang et al. [44], Kang et al. [7], Huang et al. [6], Aceng et al. [28] and Li et al. [33] are used in the analysis of encryption speed. The simulation results are shown in Table 14 and Figure 11, from which we can conclude that the proposed encryption scheme has a faster encryption speed than similar ones.

## 6. Conclusions

Firstly, a novel, improved Josephus ring-based permutation algorithm is proposed in this paper. Different from the traditional Josephus ring scrambling algorithm, IJRBP combines the advantages of the Josephus ring and chaotic mapping and replaces the remove operation with the position exchange operation, which overcomes the shortcomings of poor confusion and the long scrambling time of the existing permutation algorithms, including the TJRP. Then, based on the IJRBP, a new encryption scheme was suggested. In the developed cryptosystem, to ensure high plaintext sensitivity, a plain image is used to determine the shift step of the circular shift operation to generate the diffusion matrix. Finally, thorough experiments, including key space analyses, histogram analyses, correlation analyses, plaintext sensitivity analyses, information entropy analyses, robustness against noise analyses, data loss analyses, and encrypted time analyses are conducted, and their results prove that the proposed encryption scheme has high security and computational efficiency. In our future work, we will exploit the potential of IJRBP to expand its application scenarios while exploring other possibilities to optimize the encryption approach for better robustness.

## Figures and Tables

**Table 1:** Cryptanalysis of different schemes.

Schemes	Category	Cryptanalyzed by	Attacks Employed
Pak et al. (2017) [17]	NPR	Wang et al. (2018) [39]	CPA
Hua et al. (2018) [4]	NPR	Chen et al. (2020) [40]	CPA
Huang et al. (2018) [6]	PR	Hu et al. (2020) [41]	CPA
Zhang et al. (2016) [15]	NPR	Wu et al. (2018) [42]	CPA
Zhen et al. (2016) [26]	PR	Su et al. (2017) [43]	CPA

**Table 2:** Comparison of scrambling schemes published recently.

Schemes	Technique	Image	Speed (s)	Comments
Josephus ring with step = 3	Josephus ring	Gray	591.2136	Poor permutation effect and low efficiency
[1]	Circular shift	Gray	0.0105	High efficiency but average permutation effect
[5]	Sorting	Gray	75.2105	Better permutation effect but low efficiency
[24]	Sorting	Gray	6215.2372	Better permutation effect but unacceptable inefficiency
[18]	Improved Josephus ring	Gray	121.4508	Better permutation effect than Josephus ring but low efficiency
[6]	2D cat map	Color	2.8945	Poor permutation effect and low efficiency
[38]	Sorting	Color	2.4200	Poor permutation effect and low efficiency
proposed	IJRBP	Gray	0.1243	Excellent confusion effect and high time efficiency
proposed	IJRBP	Color	0.2223	Excellent confusion effect and high time efficiency

**Table 3:** Quantitative results of PSNR.

Gray Image (512×512)	Proposed	Ref. [44]	Ref. [7]	Ref. [28]	Ref. [33]
Lena	9.2222	9.5301	9.5244	9.2142	9.2196
Airfield	8.4518	8.4455	8.4325	8.4246	8.4496
Boat	9.2938	9.2975	9.2841	9.3047	9.2922
Ruler	4.7589	4.7686	4.7482	4.7580	4.7727
Average	7.9316	8.0104	7.9973	7.9253	7.9335

**Table 4:** Key space comparisons.

Schemes	Proposed	Ref. [44]	Ref. [45]	Ref. [46]	Ref. [28]	Ref. [47]	Ref. [48]
Key space size	$2^{170}$	$2^{512}$	$10^{98}$	$10^{15}\times 2^{256}$	$2^{170}$	$2^{159}$	$2^{198}$

**Table 5:** VIH analysis results for various schemes.

Gray Image (512×512)	Proposed	Ref. [44]	Ref. [7]	Ref. [28]	Ref. [33]
Lena	984.13	931.05	1145.87	967.85	1025.5
Airfield	1088.9	1145.07	1136.73	1077.8	940.73
Boat	1011.4	1007.90	1630.34	942.87	998.27
Ruler	885.38	995.73	6529.51	20,064	997.84
Average	992.45	1019.76	2610.61	5763.13	990.58

**Table 6:** Correlation coefficients of plain images and ciphered images.

Image	Original Image			Cipher Image		
	Horizontal	Vertical	Diagonal	Horizontal	Vertical	Diagonal
Lena	0.972	0.9853	0.9684	−0.0005	0.0000	−0.0034
Baboon	0.8666	0.7593	0.7269	0.0001	−0.0007	−0.0028
Barbara	0.8595	0.959	0.8426	−0.0018	−0.0007	0.0023
Cameraman	0.9338	0.9597	0.9074	−0.0023	0.0019	−0.0027

**Table 7:** Correlation coefficients of ciphered Lena image obtained by different algorithms.

Direction	Proposed	Ref. [45]	Ref. [7]	Ref. [15]	Ref. [46]	Ref. [28]	Ref. [33]
Horizontal	−0.0005	−0.0139	0.0025	−0.0042	0.0064	0.0015	−0.004
D	0.0000	6.7947 × 10${}^{-4}$	−0.0026	−0.0036	0.0029	−0.0034	−0.0052
V	−0.0034	0.0177	−0.0019	0.0005	0.0078	0.0051	−0.0017
Average	0.0013	0.0107	0.0023	0.0027	0.0057	0.0033	0.0024

**Table 8:** Correlation coefficients of ciphered Baboon image obtained by different algorithms.

Direction	Proposed	Ref. [45]	Ref. [7]	Ref. [15]	Ref. [46]	Ref. [28]	Ref. [33]
Horizontal	0.0001	−0.0106	0.0019	0.0021	0.0018	−0.0041	0.0002
D	−0.0007	0.0180	0.0036	0.0023	0.0056	−0.0043	−0.0026
V	−0.0028	0.0036	0.0014	0.0012	−0.0016	0.0003	0.0029
Average	0.0012	0.0072	0.0023	0.0018	0.0030	0.0029	0.0019

**Table 9:** Key sensitivity test results using NPCR and UACI.

Image	Index	$K_{1}+10^{-14}$	$K_{2}+10^{-14}$	$K_{3}+10^{-14}$	$N_{0}+1$	Theoretical Values
Lena	NPCR	99.6098	99.6087	99.6087	99.6096	99.6094
	UACI	33.4580	33.4658	33.4632	33.4642	33.4635
Baboon	NPCR	99.6055	99.6088	99.6075	99.6096	99.6094
	UACI	33.4342	33.4658	33.4691	33.4667	33.4635
Boat	NPCR	99.6067	99.6081	99.6092	99.6084	99.6094
	UACI	33.4395	33.4699	33.4644	33.4649	33.4635
Barbara	NPCR	99.6088	99.6085	99.6096	99.6095	99.6094
	UACI	33.4613	33.4692	33.4659	33.4662	33.4635

**Table 10:** Plaintext sensitivity test results using NPCR and UACI.

Image	Type	Size	NPCR (99.6094)	UACI (33.4635)
Lena	gray	512×512	99.6095	33.4647
Dollar	gray	512×512	99.6099	33.4633
Boat	gray	512×512	99.6089	33.4673
Plane	gray	512×512	99.6095	33.4658
Barbara	gray	512×512	99.6088	33.4637
Baboon	gray	512×512	99.6093	33.4633
Lena	gray	256×256	99.6093	33.4647
Cameraman	gray	256×256	99.6071	33.4766
Lena in Ref. [45]	gray	512×512	99.58	33.43
Baboon in Ref. [45]	gray	512×512	99.63	33.41
Cameraman in Ref. [45]	gray	256×256	99.61	33.46
Lena in Ref. [7]	gray	512×512	99.6178	33.4412
Baboon in Ref. [7]	gray	512×512	99.6004	33.4522
Cameraman in Ref. [7]	gray	256×256	99.5987	33.4316
Lena in Ref. [15]	gray	512×512	99.6155	33.4988
Lena in Ref. [46]	gray	512×512	99.5994	33.4647
Baboon in Ref. [46]	gray	512×512	99.6351	33.4857
Lena in Ref. [28]	gray	512×512	0.0003	0.0015
Baboon in Ref. [28]	gray	512×512	0.0003	0.0015
Lena in Ref. [33]	gray	512×512	0.0003	0.0015
Baboon in Ref. [33]	gray	512×512	0.0003	0.0015

**Table 11:** The NPCR and UACI results of special images.

Index	P1	P2	P3	P4	Theoretical Values
NPCR	99.6090	99.6108	99.6093	99.6099	99.6094
UACI	33.4607	33.4636	33.4663	33.4677	33.4635

**Table 12:** Information entropy calculation results.

Image	Type	Size	Plain Image	Cipher Image
Lena	gray	512×512	7.4474	7.9993
Baboon	gray	512×512	7.1391	7.9993
Barbara	gray	512×512	7.4664	7.9993
Lena in Ref. [45]	gray	512×512	7.4456	7.9993
Baboon in Ref. [45]	gray	512×512	7.3579	7.9994
Lena in Ref. [7]	gray	512×512	7.4455	7.9993
Baboon in Ref. [7]	gray	512×512	7.3585	7.9993
Lena in Ref. [15]	gray	512×512	–	7.9992
Baboon in Ref. [15]	gray	512×512	–	7.9992
Lena in Ref. [46]	gray	512×512	–	7.9993
Baboon in Ref. [46]	gray	512×512	–	7.9992
Lena in Ref. [28]	gray	512×512	7.4474	7.9993
Baboon in Ref. [28]	gray	512×512	7.1391	7.9993
Lena in Ref. [33]	gray	512×512	7.4474	7.9994
Baboon in Ref. [33]	gray	512×512	7.1391	7.9994

**Table 13:** PNSR analysis of noise attacks and data loss.

Noise Attacks or Data Loss	PSNR
Salt-and-pepper noise (0.1)	19.2388
Salt-and-pepper noise (0.2)	16.2361
Salt-and-pepper noise (0.3)	14.4661
Salt-and-pepper noise (0.4)	13.2087
Data loss (60:250,60:250)	17.8117
Data loss (1:100,1:512)	16.2678
Data loss (1:512,250:450)	13.2774
Data loss (300:512,1:512)	13.0381

**Table 14:** Execution speed for cryptographic systems (seconds).

Size	Gray Image (Lena)					
	Proposed	Ref. [44]	Ref. [7]	Ref. [6]	Ref. [28]	Ref. [33]
512×512	0.2398	4.3778	0.9703	1.2723	0.1236	22.2586
256×256	0.0565	1.0886	0.2612	0.3727	0.0301	5.6697
128×128	0.0119	0.3121	0.0712	0.0866	0.0076	1.3892

## Data Availability

Not applicable.
